# Reply to Compton et al.: Another paradoxical misunderstanding

**DOI:** 10.1073/pnas.2536185123

**Published:** 2026-02-02

**Authors:** George Butler, Joanna Baker, Sarah R. Amend, Kenneth J. Pienta, Chris Venditti

**Affiliations:** ^a^University College London Cancer Institute, University College London, London WC1E 6DD, United Kingdom; ^b^Cancer Ecology Center, The Brady Urological Institute, Johns Hopkins School of Medicine, Baltimore, MD 21287; ^c^School of Biological Sciences, University of Reading, Reading RG6 6AS, United Kingdom; ^d^School of Life, Health, and Chemical Sciences, The Open University, Milton Keynes MK7 6AA, United Kingdom

Phylogenetic statistical methods provide an opportunity to formally test the relationship between phenotypic traits (1). When used correctly, phylogenetic models offer powerful insight into the emergence of adaptive trends across evolutionary time (2). Unfortunately, Compton et al. (3) demonstrate how methodological misunderstandings can lead to incorrect interpretations and inaccurate conclusions.

Compton et al. (3) suggest that modeling tumor prevalence (count data) via a Poisson regression model as a function of the number of necropsies, and other covariates, implies that all species have the same rate of cancer. The claim that by instead modeling tumor prevalence divided by the number of necropsies as a proportion, it is possible to account for *different intrinsic rates of cancer*. However, not only does this approach violate the underlying assumptions of phylogenetic generalized least squares (PGLS) (4), but it also fails to capture variation in intrinsic cancer rates as Compton et al. suggest (3). Note that this is the case even after arcsine transformation—which is widely recognized as inappropriate for analyzing proportional data even in nonphylogenetic context (5). PGLS assumes Brownian-motion trait evolution with a single estimated variance (σ^2^), implying a shared evolution rate across all species (6, 7). Because Compton et al. use such a model (3), their claim that their approach captures difference in intrinsic cancer rates lacks methodological support.

To study tumor prevalence as a proportion in the way Compton et al have done previously (8), it would be most appropriate to use a binomial or beta-binomial regression framework (9). Indeed, when we use a phylogenetic beta-binomial regression on the proportion data, we recover results consistent with Butler et al. (2) (Table 1), confirming that the conclusions are robust to whether tumor prevalence is modeled as counts or proportions.

**Table 1.** Comparing Poisson and Beta-binomial models

Model	Growth	Body mass	Path-wise rate	Lineage diversification—Birds	Lineage diversification—Mammals
Poisson	Benign	β = 0.140, P_x_ = 0.001	β = −0.210, P_x_ = 0.230	β = 0.554, P_x_ = 0.027	β = −0.040, P_x_ = 0.415
Poisson	Malignant	β = 0.172, P_x_ = 0.002	β = −0.944, P_x_ = 0.002	β = 0.667, P_x_ = 0.022	β = −0.041, P_x_ = 0.426
Beta-binomial	Benign	β = 0.151, P_x_ = 0.001	β = −0.181, P_x_ = 0.286	β = 0.615, P_x_ = 0.032	β = −0.027, P_x_ = 0.442
Beta-binomial	Malignant	β = 0.193, P_x_ = 0	β = −1.031, P_x_ = 0.007	β = 0.695, P_x_ = 0.043	β = −0.053, P_x_ = 0.416

Parameter estimates (β) and significance (Px) are reported, with significant covariates highlighted in red. Significance was assessed by the proportion of the posterior distribution crossing zero (Px < 0.05). In the Poisson model, benign and malignant tumor prevalence depended on the number of necropsies, body mass, path-wise rate, and lineage diversification, whereas the Beta-binomial model evaluated the effect of body mass, path-wise rate, and lineage diversification. Both models estimated shared slopes across birds and mammals for body mass and path-wise rate but class-specific slopes for lineage diversification. Qualitative results were consistent across models.

Compton et al.’s response (3) implies that they are considering a series of multiple pairwise regressions with each covariate separately as equivalent to a multiple regression analysis. However, unlike such pairwise comparisons, in a multiple regression model, the effects of body size, rate of body size evolution, and lineage diversification on tumor prevalence are estimated simultaneously. This apparent misunderstanding of the type of multiple regression analysis utilized by Butler et al. (2) is further illustrated by Compton et al.’s (3) inaccurate assertion that predictors “add or subtract a fixed number.” In multiple regression models, the effect of each covariate is estimated conditional on all other covariates; for example, the body size effect is estimated conditional on the rate of body size evolution. This conditional interpretation is a core strength of multiple regression and allows multiple hypotheses to be evaluated simultaneously. As a result, interaction terms between necropsy number and other covariates are neither required nor meaningful, because necropsy number enters the model as sampling effort rather than as a biological predictor.

We are pleased that Compton et al. agree that our findings are *important additions to the study of comparative oncology* (3). However, this exchange highlights the necessity of aligning statistical models with the structure of the data and the biological questions at hand, rather than assuming that one-size-fits-all methods that risk obscuring meaningful evoution signal.

## References

[1] M. Pagel, Inferring the historical patterns of biological evolution. Nature **401**, 877–884 (1999). 10553904 10.1038/44766

[2] G. Butler, J. Baker, S. R. Amend, K. J. Pienta, C. Venditti, Divergent evolutionary dynamics of benign and malignant tumors. Proc. Natl. Acad. Sci. U.S.A. **122**, e2519203122 (2025). 41196351 10.1073/pnas.2519203122 PMC12625976

[3] Z. T. Compton , Divergent understandings in comparative oncology. Proc. Natl. Acad. Sci. U.S.A. **123**, e2532925123. (2025).10.1073/pnas.253292512341628321

[4] V. J. Lynch, Peto’s paradox revisited (revisited, revisited, revisited, and revisited yet again). Proc. Natl. Acad. Sci. U.S.A. **122**, e2502696122 (2025). 40163737 10.1073/pnas.2502696122 PMC12002202

[5] D. I. Warton, F. K. C. Hui, The arcsine is asinine: The analysis of proportions in ecology. Ecology **92**, 3–10 (2011). 21560670 10.1890/10-0340.1

[6] C. Venditti, A. Meade, M. Pagel, Multiple routes to mammalian diversity. Nature **479**, 393–396 (2011). 22012260 10.1038/nature10516

[7] G. Butler, J. Baker, S. R. Amend, K. J. Pienta, C. Venditti, No evidence for Peto’s paradox in terrestrial vertebrates. Proc. Natl. Acad. Sci. U.S.A. **122**, e2422861122 (2025). 39993196 10.1073/pnas.2422861122 PMC11892590

[8] Z. T. Compton , Cancer prevalence across vertebrates. Cancer Discov. **15**, 227–244 (2025). 39445720 10.1158/2159-8290.CD-24-0573 PMC11726020

[9] J. C. Douma, J. T. Weedon, Analysing continuous proportions in ecology and evolution: A practical introduction to beta and dirichlet regression. Methods Ecol. Evol. **10**, 1412–1430 (2019).

